# Lineage-specific serology confirms Brazilian Atlantic forest lion tamarins, *Leontopithecus chrysomelas* and *Leontopithecus rosalia*, as reservoir hosts of *Trypanosoma cruzi* II (TcII)

**DOI:** 10.1186/s13071-016-1873-y

**Published:** 2016-11-15

**Authors:** Charlotte L. Kerr, Tapan Bhattacharyya, Samanta C. C. Xavier, Juliana H. Barros, Valdirene S. Lima, Ana M. Jansen, Michael A. Miles

**Affiliations:** 1 Faculty of Infectious and Tropical Diseases, London School of Hygiene and Tropical Medicine, Keppel St, London, UK; 2 Laboratory of Trypanosomatid Biology, Oswaldo Cruz Institute, Fiocruz, Av. Brasil 4365, Rio de Janeiro, RJ Zip Code 21040-360 Brazil

**Keywords:** *Trypanosoma cruzi*, ELISA, Serology, Lineage-specific, Primates, Brazil, Chagas disease

## Abstract

**Background:**

*Trypanosoma cruzi*, the agent of Chagas disease in humans, has a vast reservoir of mammalian hosts in the Americas, and is classified into six genetic lineages, TcI-TcVI, with a possible seventh, TcBat. Elucidating enzootic cycles of the different lineages is important for understanding the ecology of this parasite, the emergence of new outbreaks of Chagas disease and for guiding control strategies. Direct lineage identification by genotyping is hampered by limitations of parasite isolation and culture. An indirect method is to identify lineage-specific serological reactions in infected individuals; here we describe its application with sylvatic Brazilian primates.

**Methods:**

Synthetic peptides representing lineage-specific epitopes of the *T. cruzi* surface protein TSSA were used in ELISA with sera from Atlantic Forest *Leontopithecus chrysomelas* (golden-headed lion tamarin), *L. rosalia* (golden lion tamarin), Amazonian *Sapajus libidinosus* (black-striped capuchin) and *Alouatta belzebul* (red-handed howler monkey).

**Results:**

The epitope common to lineages TcII, TcV and TcVI was recognised by sera from 15 of 26 *L. chrysomelas* and 8 of 13 *L. rosalia*. For 12 of these serologically identified TcII infections, the identity of the lineage infection was confirmed by genotyping *T. cruzi* isolates. Of the TcII/TcV/TcVI positive sera 12 of the 15 *L. chrysomelas* and 2 of the 8 *L. rosalia* also reacted with the specific epitope restricted to TcV and TcVI. Sera from one of six *S. libidinous* recognised the TcIV/TcIII epitopes.

**Conclusions:**

This lineage-specific serological surveillance has verified that Atlantic Forest primates are reservoir hosts of at least TcII, and probably TcV and TcVI, commonly associated with severe Chagas disease in the southern cone region of South America. With appropriate reagents, this novel methodology is readily applicable to a wide range of mammal species and reservoir host discovery.

## Background

*Trypanosoma cruzi* is the causative agent of Chagas disease, regarded as the most important parasitic disease in Latin America with a burden of 0.67 million disability-adjusted life years (DALY) [1, 2]. *Trypanosoma cruzi* has a complex system of domestic, peridomestic and sylvatic transmission cycles involving mammals, triatomines and humans, some of which overlap and may interact, some of which may be entirely disparate. Enzootic in sylvatic mammalian species from southern states of the USA to Southern Argentina, *T. cruzi* is found in seven orders and a huge range of species representing a vast reservoir for the parasite [3, 4].

*Trypanosoma cruzi* exhibits great genetic diversity and is currently classified into six distinct genetic lineages, TcI-VI, with a possible seventh, TcBat [5–7]. It has been suggested that the distribution of the distinct lineages is associated with different geographies, ecosystems, mammalian hosts and even clinical presentation of the human disease. However current associations are restricted and fragmentary due to both the scarce sampling in some settings and the difficulty in isolating and genotyping *T. cruzi* from sylvatic mammals, potentially resulting in an oversimplification or misinterpretation of these lineage-specific findings [7–10]. The parasite may be identified in blood films in the acute phase but is very rarely found in peripheral blood in the chronic phase and so haemocultures, xenodiagnoses or amplification of DNA by PCR from blood may be applied, with low sensitivity.

TcI is the most widespread recorded lineage, both geographically and in sylvatic mammalian species, while TcII is common in the domestic cycle [7, 9]. Nevertheless, TcII is increasingly reported in sylvatic mammals in Southern American biomes, particularly in the Brazilian Atlantic Forest [4, 7]. Jansen et al. [5] found 58% of *T. cruzi* isolates from Brazilian sylvatic mammals to be TcI while 17% were TcII. TcIII is seldom isolated from humans, whereas TcIV is a secondary cause of Chagas disease in Venezuela and in the Brazilian Amazon region [7, 11, 12]. TcV and TcVI are relatively recent hybrids of TcII and TcIII and along with TcII have largely been associated with the domestic cycle, while sylvatic hosts are presently not clearly defined [7, 11, 13, 14]. It is particularly desirable to identify the mammalian reservoir of these lineages in the Southern Cone region due to their implication in severe human disease in the form of megacolon, megaoesophagus and chagasic cardiomyopathy [7, 13].

As the epidemiological picture of Chagas disease in Latin America shifts, the sylvatic reservoir becomes increasingly important. Currently an estimated 8 million people are affected in 21 countries [1, 15]. However, the distribution is changing with migration both from rural to urban areas and from endemic to non-endemic countries [15, 16]. Domestic transmission by *Triatoma infestans* was officially eliminated in Brazil in 2006 [16–18]. Cases of oral transmission are now more frequently observed, and sporadic cases arising from sylvatic adult triatomines entering dwellings [16, 19–22]. Of more than 1,000 acute infections of Chagas disease reported in Brazil during 2000–2010, 776 (71%) were considered to have been acquired by the oral route from contaminated juices or foods, the majority in the Amazon region of Brazil [21, 23–25].

Knowledge of the vector, host and geography of individual lineages is limited by the difficulty in isolating the parasite, which sequesters in host tissue. Currently several serological assays are in use, however these do not give any insight into the specific infecting lineages and as such an indirect method that can identify lineage-specific infection history is desirable in order to elucidate the ecological cycles of the lineages and aid discovery of novel mammalian reservoirs. [20]. Lineage-specific serology requires an antigen sufficiently polymorphic both at the genetic locus and in the amino acid sequence produced. The Trypomastigote Small Surface Antigen (TSSA), a surface protein of the bloodstream trypomastigote, was originally identified as having two alleles corresponding to the lineages TcI and TcII (which at that time encompassed TcII-VI) and was the first serological marker to identify the lineage of human infection [26]. Chagasic patients were only TSSA-II seropositive, which led to the suggestion that TcI could be benign. However, this suggestion was in conflict with the geographical predominance of TcI and occurrence of chagasic cardiomyopathy north of the Amazon [10, 27]. Further molecular analysis of TSSA by Bhattacharyya et al. [28] demonstrated much greater diversity at this locus, with an increased number of lineage-specific epitopes, implying potential for serology that can differentiate between lineages. The sequence previously described as being shared by TcII-VI was shown to be restricted in distribution to TcII, TcV and TcVI, whilst TcIII and TcIV each have their own distinct lineage-specific epitope, although only differing from each other by two amino acids. The TSSA locus is heterozygous in the hybrid lineages TcV and TcVI and encodes for a further TSSA peptide that they share, differing from the TcII epitope by a single amino acid substitution [11, 28]. BLAST searches against available *T. cruzi* sequences have confirmed that these epitopes are conserved within their lineages, and furthermore, comprehensive searches have not detected the epitopes in any other organisms. Synthetic peptides representing lineage-specific epitopes have been used in an ELISA to identify infecting lineages in human chagasic sera from a range of endemic countries, demonstrating a geographical and clinical variation in epitope recognition [11]. However, the TSSApep-I gave no clear specific reaction with human chagasic sera from TcI endemic regions, potentially due to low immunogenicity or a conformation of the peptide differing from that of the native antigen. Cimino et al. [29] have published the only study on lineage-specific serology in naturally infected mammals, demonstrating positive results with an ELISA using *E. coli* produced recombinant TSSA-II antigen (the epitope shared by TcII, TcV and TcVI) when applied to Argentinian canine sera. Bhattacharyya et al. [13] have also applied an expanded range of TSSA epitopes in an ELISA with synthetic lineage-specific peptides and experimentally infected mouse sera, with a pilot study of a small range of sylvatic Brazilian primates.

Here we have applied a serological test using lineage specific TSSA epitopes to the sera of Brazilian primates from the Atlantic Forest and Amazon regions, in order to identify the historic infecting lineage(s) and further expand knowledge of transmission cycles in these biomes.

## Methods

### Primate sera

**Fig. 1:**
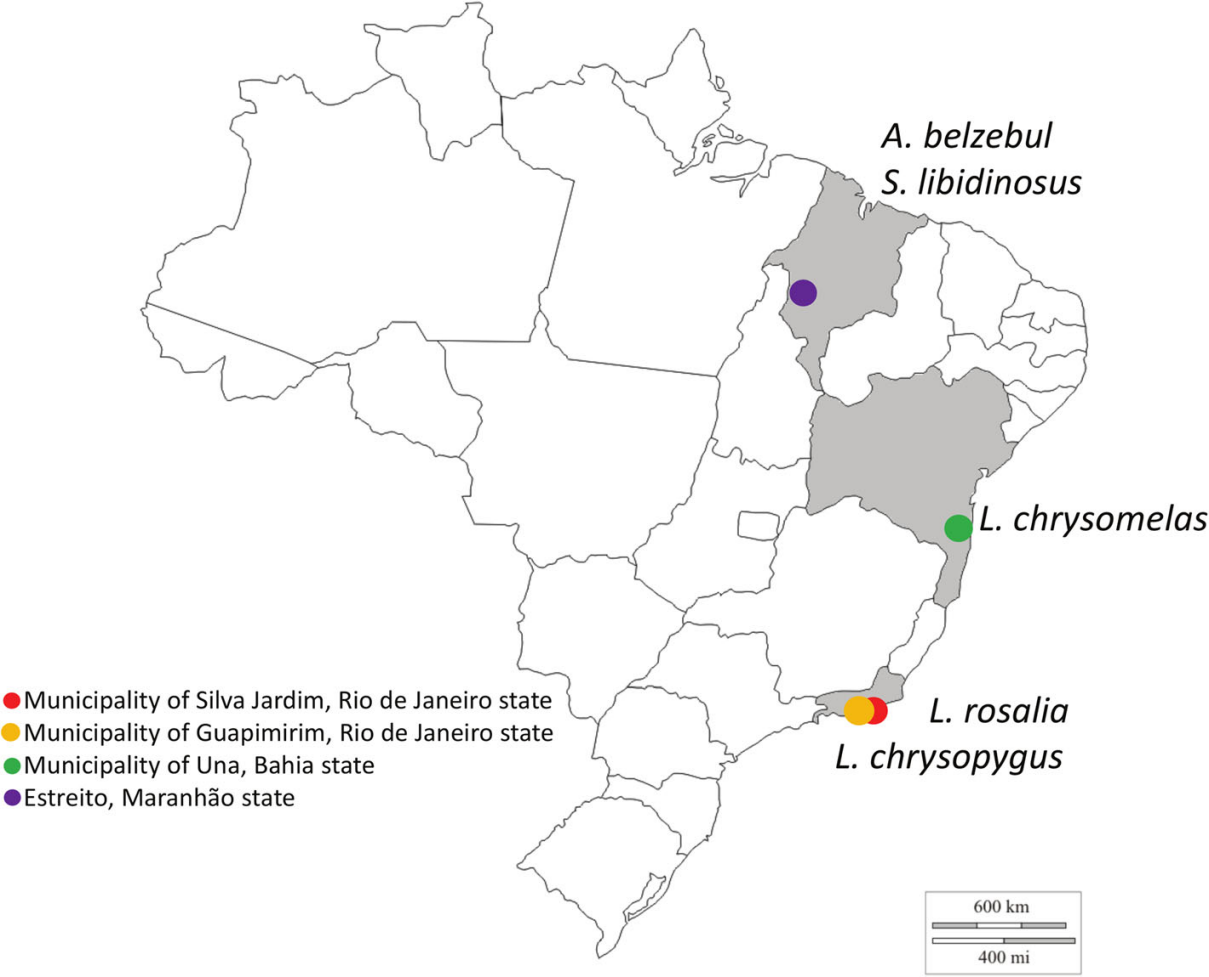
Map of Brazil showing the origins of primate samples tested using the lineage-specific ELISA (http://d-maps.com/carte.php?num_car=24873&lang=en)

Archived sera were used from the cryobanks of Fiocruz, Rio de Janeiro, which had been obtained from ongoing field research. The following species were tested: *Leontopithecus rosalia* (golden lion tamarin) (*n* = 16) of Silva Jardim municipality, Rio de Janeiro State, *Leontopithecus chrysopygus* (black lion tamarin) (*n* = 1) of Guapimirim in Rio de Janeiro State, *Leontopithecus chrysomelas* (golden-headed lion tamarin) (*n* = 33) of Una municipality in Bahia, *Alouatta belzebul* (red-handed howler monkey) (*n* = 8) of Estreito, Maranhão State and *Sapajus libidinosus* (black-striped capuchin) (*n* = 12) of Estreito, Maranhão State. The geographical origins of these animals are shown in Fig. 1. Haemoculture had been performed on all samples and as far as possible lineage of isolates obtained had been characterised by multiplex polymerase chain reaction (PCR) of the mini-exon region. This differentiates between TcI, TcII (TcII/V/VI), TcIII/IV and *Trypanosoma rangeli* [8]. The indirect fluorescent antibody test (IFAT) was performed on all samples. Nine of the *L. rosalia* samples have been further characterised using polymerase chain reaction: restriction fragment length polymorphisms (PCR: RFLP), by single nucleotide polymorphisms (SNPs) in the *HSP60* and *GPI* loci and by PCR amplification of the D7 divergent domain of the 24Sα rRNA gene (LSU rDNA), as described by Lewis et al. [30].

### TSSA lineage-specific peptides

**Fig. 2:**
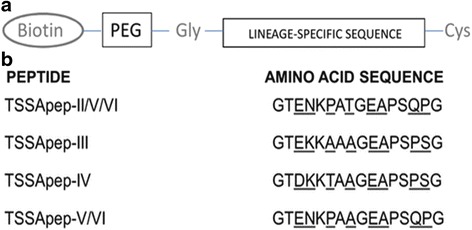
*Trypanosoma cruzi* lineage-specific peptides (TSSApep) used in primate serology. **a** The components of the peptides synthesised: N-terminal biotinylation; PEG spacer; Gly; the lineage-specific sequence; C-terminal Cys. **b** The amino acid sequences of the lineage-specific TSSA epitopes in the synthetic peptides (TSSApep-). Polymorphic residues are underlined. *Abbreviations*: Cys, cysteine residue; Gly, glycine residue; PEG, polyethylene glycol

Peptides TSSApep-II/V/VI, TSSApep-III, TSSApep-IV, and TSSApep-V/VI, representing epitopes found in those *T. cruzi* lineages, were synthesised with N-terminal biotinylation. As shown in Fig. 2, these peptides differ by crucial lineage-specific amino acids. As described above, despite extensive application, TSSApep-I, representing the TcI TSSA epitope, has rarely been recognised in ELISA and thus was not used here. Details of the peptide synthesis and bioinformatic analysis of their antigenicity have been described previously [11].

### Lineage-specific ELISA

Flat bottomed ELISA strip plates (767071: Greiner Bio-one) were coated with 1 μg /100 μl/well of avidin (A9275: Sigma-Aldrich, Gillingham, UK) diluted in 1× carbonate-bicarbonate coating buffer (15 mM Na_2_CO_3_, 34 mM NaHCO_3_, pH 9.6) for binding to biotinylated lineage-specific TSSApep, and separate wells were coated directly with Y strain *T. cruzi* lysate (Biomanghuinos, Fiocruz, RJ) at 0.2 μg /100 μl/well as a serological control. Plates were covered with an adhesive sheet and incubated overnight at 4 °C. The following day, unbound avidin and lysate were removed, the wells washed three times with PBS containing 0.05% (vol/vol) Tween 20 (P7949: Sigma-Aldrich) (PBS/T), then wells were blocked with 200 μl blocking buffer [PBS/2% skimmed milk powder (Premier International Foods, Spalding, UK)] at 37 °C for 2 h. Following three washes, 1 μg/100 μl/well of lineage-specific TSSApep in PBS/T containing 2% skimmed milk powder (PBS/T/M) was incubated with the avidin-coated wells at 37 °C for 1 h. For each sample, one avidin-coated well remained without peptide as a no-peptide control. Following three washes, 100 μl/well of a 1:200 dilution of primate serum in PBS/T/M was applied and incubated at 37 °C for 1 h. Following six washes, 100 μl/well of goat anti-human IgG-HRP (A0170: Sigma-Aldrich) diluted 1:10,000 in PBS/T/M was added, and incubated at 37 °C for 1 h. The wells were then washed 6 times and developed by applying 100 μl/well of 3,3′,5,5′-Tetramethylbenzidine (Biomanguinhos Fiocruz, RJ), and incubated in the dark for 10–15 min at room temperature. The reaction was stopped by adding 50 μl/well of 2 M H_2_SO_4_ and the absorbance values were then read at 450 nm. Replica plates were run simultaneously.

### Statistical analysis

Cut-off values for ELISAs were calculated from the mean plus 3 standard deviations compared to negative controls. The same negative sylvatic animal was used as a control on every plate.

## Results

### TSSApep serology identifies hosts of specific *T. cruzi* lineages

**Fig. 3:**
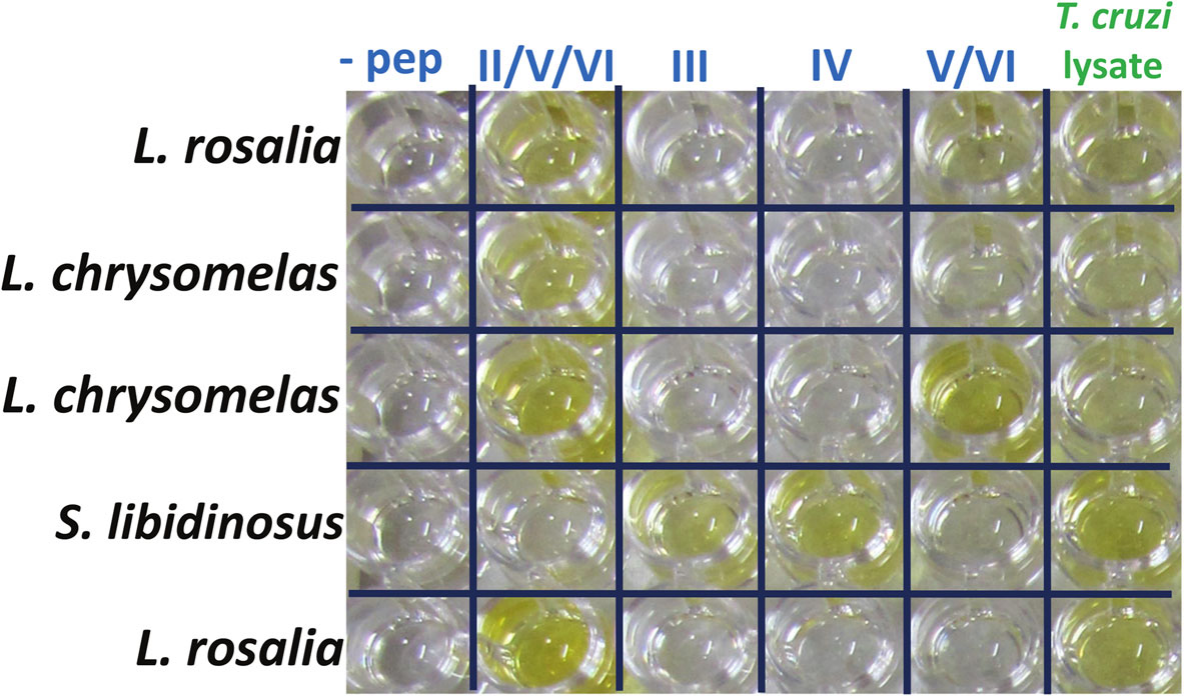
TSSApep lineage-specific ELISA in primates. The plate shows both TSSApep-II/V/VI only and TSSApep-II/V/VI and TSSApep-V/VI reactions in *L. rosalia* samples, TSSApep-II/V/VI and TSSApep-V/VI reactions in *L. chrysomelas* and a TSSApep-III and TSSApep-IV reaction in *S. libidinosus* samples

**Fig. 4:**
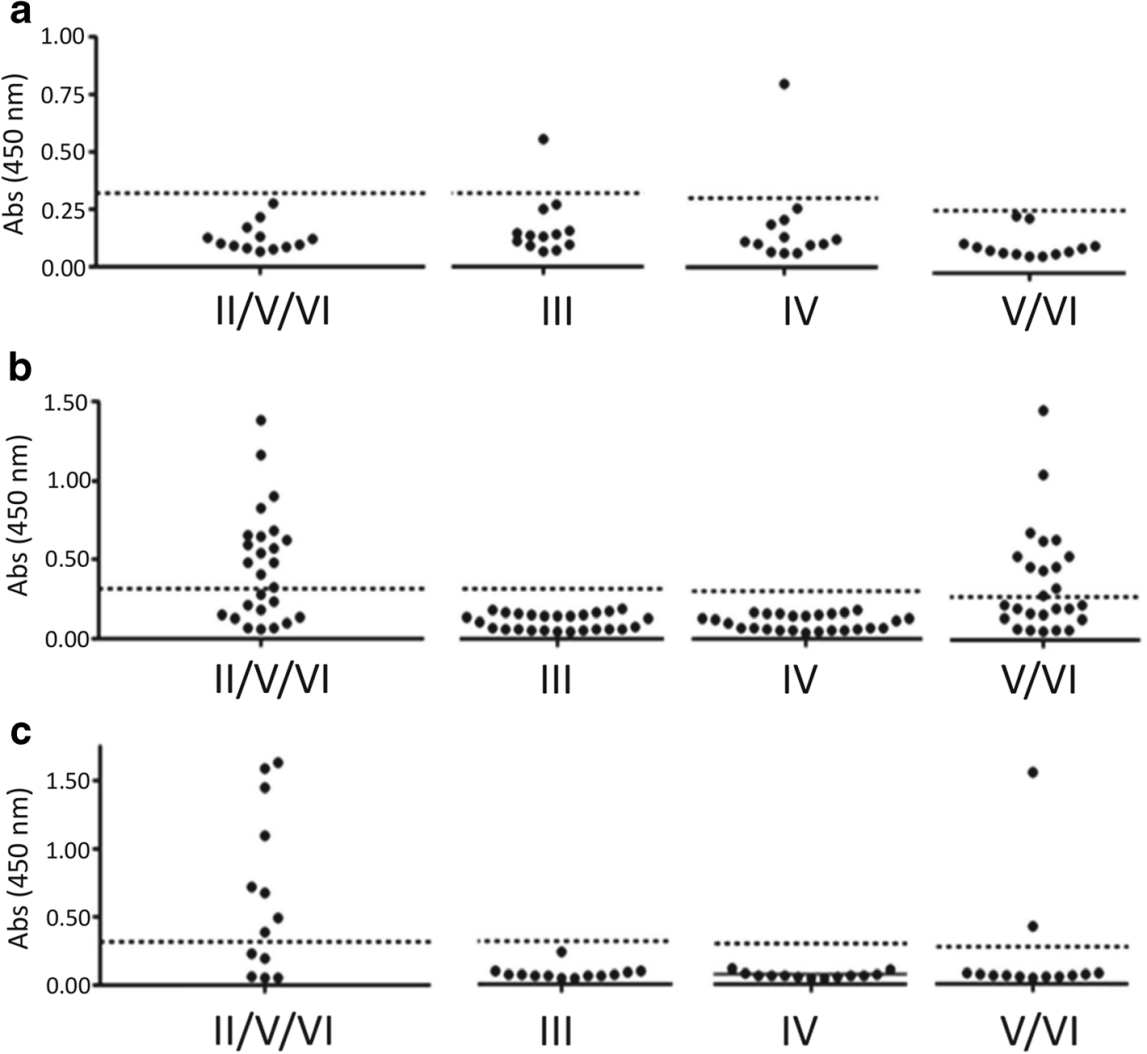
Distribution of lineage-specific TSSApep recognition. Individual lineage-specific ELISA results plotted as single data points (mean of duplicate absorbance values read at 450 nm) for: **a** Amazonian primates, **b** *L. chrysomelas*, and **c** *L. rosalia*. The dotted line represents the negative cut-off point for each peptide

Figure 3 shows an example of *T. cruzi* lineage-specific ELISA using primate sera, demonstrating the specific nature of the TSSApep recognition. Table 1 and Fig. 4 summarise the ELISA results with the primate samples. Table 1 gives the peptide results in seropositive primates tested by species, including the previous haemoculture test results, while Fig. 4 presents the mean absorbance value for each lineage-specific peptide for every seropositive animal tested. As observed previously with human samples, a small proportion of the primate samples, across four different species reacted non-specifically with avidin [11]. Such primate samples were excluded from the analysis and were not included in Table 1 or Fig. 4.

**Table 1:** Comparison of TSSApep ELISA reactions with haemoculture and genotyping data. All samples had tested positive for *T. cruzi* infection with IFAT. Seven samples reacted non-specifically and are excluded (see text)

Species	Biome	Region	Haemoculture	Genotyping^a^	n^b^	TSSA peptide reaction				
						II/V/VI	III	IV	V/VI	Lysate^c^
*L. chrysomelas*	Atlantic Rainforest	Una, Bahia	Positive	I	1	1	0	0	1/1 of TSSA II/V/VI	0
				II	11	8	0	0	6/8 of TSSA II/V/VI	5 (1)
				Uncharacterised	6	4	0	0	4/4 of TSSA II/V/VI	3 (1)
			Negative		8	2	0	0	1/2 of TSSA II/V/VI	5 (4)
*L. chrysopygus*	Atlantic Rainforest	Guapimirim, RJ	Positive	II	1	0	0	0	0	0
*L. rosalia*	Atlantic Rainforest	Silva Jardim, RJ	Positive	II	8 (4)	3	0	0	1/3 of TSSA II/V/VI	2
				Mixed I/II	1 (1)	1	0	0	1/1 of TSSA II/V/VI	1
				Uncharacterised	1	1	0	0	0	1
			Negative		3	3	0	0	0	3
*S. libidinosus*	Amazon	Estreito, Maranhão	Positive	Uncharacterised	2	0	1^d^	1^d^	0	1^d^
			Negative		4	0	0	0	0	0
*A. bezebul*	Amazon	Estreito, Maranhão	Positive	Mixed I/IV	1	0	0	0	0	0
				Uncharacterised	1	0	0	0	0	1 (1)
			Negative		5	0	0	0	0	0

^a^All samples genotyped by PCR of the mini-exon

^b^Parentheses indicate TcII infections confirmed by PCR:RFLP based on SNPs in the *HSP60* and *GPI* loci and by PCR of LSU rDNA

^c^Parentheses indicate samples that reacted with lysate only

^d^Same sample reacting with TSSApep-III and TSSApep-IV and lysate

Sera from primates from both the Amazonian region and the Atlantic Forest recognised lineage-specific peptides. The majority of peptide reactions were to TSSApep-II/V/VI (23/53 animals, excluding non-specific reaction), the epitope present in TcII, TcV and TcVI. Figure 3 provides examples of this peptide recognition by both *L. rosalia* and *L. chrysomelas* sera, indicative of infection with TcII, TcV or TcVI. Of those animals that had isolates genotyped as TcII by mini-exon typing (TcII, TcV or TcVI) a high proportion reacted with the epitope common to TcII, TcV and TcVI (11/20 excluding non-specific reactors) while seven of these 11 also reacted with TSSApep-V/VI, representing infection with a hybrid lineage TcV or TcVI. Only one Amazonian primate reacted with lineage-specific peptides, this being the *S. libidinosus* sampled in the Estreito municipality, in the Amazon region. This animal responded to both TSSApep-III and TSSApep-IV, which differ by only 2 of 16 residues, which is likely due to cross-reaction between these epitopes. The majority of Amazonian primates tested did not react with peptides or lysate and any IFAT titres were low, ranging from 1/10 to 1/40, whereas the sample that produced peptide reactions had a titre of 1/320. However, in the other species tested, *L. rosalia* and *L. chrysomelas*, titre value appeared to show no correlation with the presence or absence of peptide reactions, with reactions present in samples with IFAT titres ranging from 1/20 to 1/320 in *L. chrysomelas* and from 1/40 to 1/160 in *L. rosalia*. Peptide reactions were produced regardless of a negative or positive haemoculture result in these species. Samples from *L. chrysomelas* from Una, Bahia State, which reacted with TSSApep-II/V/VI were much more likely to also give a reaction to the TcV and TcVI restricted epitope in comparison to those of *L. rosalia* animals [80% (12/15) and 25% (2/8), respectively] from Silva Jardim, Rio de Janeiro State (Fig. 4). This indicates hybrid lineage infection in both species, potentially in conjunction with TcII infection. However, reaction to solely TSSApep-V/VI in the absence of recognition of TSSApep-II/V/VI was not seen, in accord with observation on human sera [11]. The *L. rosalia* samples were obtained over the period January 1996 to July 2005, while the *L. chrysomelas* samples tested were obtained between January 2003 and July 2005, with no apparent temporal aggregation of response to peptides.

Both the one sample that had been previously identified as TcI by mini-exon assay and that diagnosed as a mixed infection of TcI/II gave a positive reaction with TSSApep-II/V/VI and TSSApep-V/VI suggestive of mixed infections not isolated in haemoculture. Five samples that had negative haemoculture and IFAT results previously were also tested and of these one *L. chrysomelas* sample gave a positive reaction with TSSApep-II/V/VI, TSSApep-V/VI and the lysate (these results are not included in Table 1). This emphasises the importance of the application of multiple tests in order to ascertain as much information as possible from the limited available samples.

## Discussion

In Brazil Chagas disease has been largely attributed to TcII in the southern regions, while in Amazonia it has a unique epidemiology with sporadic disease largely attributed to TcI, and to a lesser extent TcIII and TcIV. TcII, TcV and TcVI have rarely been isolated in Amazonia, in contrast to endemic regions of Brazil where they predominate [3, 8].

Previous studies of the tamarins of the Atlantic Forest have shown a variable distribution of infection by the different *T. cruzi* lineages, with TcI and TcII predominating in different studies [31–36]. In particular, Lisboa et al. [36], in a study of the Atlantic rainforest mammals, found TcII to be present only in tamarins while TcI was found in both *Didelphis aurita* (big-eared opossum) and lion tamarins, with TcI predominating overall.

In the Amazon region, past research has largely demonstrated TcI infection in primates, corresponding with the principal agent of Chagas disease in humans in this region [27, 31, 35]. More recently Marcili et al. [3] isolated both TcI and TcIV (then known as TcIIa) from Amazonian primates. In that study there was a higher prevalence of TcI than TcIV corresponding to the prevalence seen in local *Rhodnius* species of triatomines and humans with orally acquired *T. cruzi* infection [3].

This study showed lineage-specific reactions in primates both from the Atlantic Forest and the Amazon region. Both peptides TSSApep-II/V/VI and TSSApep-V/VI gave lineage-specific reactions with samples from the Atlantic Forest. Sera of *L. rosalia* animals from Silva Jardim in Rio de Janeiro State much more likely to react solely with TSSApep-II/V/VI (75% of reactions, 6/8) whilst *L. chrysomelas* from Una in Bahia State were more likely to recognise both peptides (80%, 12/15). This confirms circulation of TcII in lion tamarin populations in both regions of Atlantic Forest sampled, while hybrid lineages are also circulating in both species but to a greater extent in golden-headed lion tamarins of Bahia State. The Atlantic Forest is increasingly fragmented, with the majority of fragments less than 50 hectares in area and less than 12% of the original forest remaining, with a consequent fragmentation of the wild population of these species, potentially favouring the establishment and maintenance of such distinct transmission cycles [37]. The behavioural patterns of lion tamarins are also likely to contribute to distinct transmission cycles in small populations, including their tendency to live in small family groups, occupy stable territories and sleep in tree holes [34]. As such each piece of this fragmented population must be considered as a separate unit in a much larger, complex web of transmission cycles [34]. Triatomines have been found to infest the nests of coatis, using multiple feeding sources including the coatis themselves; this may also occur in the tree holes of primates, with the primates also feeding on the bugs [38]. To the authors’ knowledge, the only isolates of these hybrid lineages in Brazil have been from a dog in the Amazon region as well as a sylvatic rodent, *Trichomys laurentius*, and two opossums in the Caatinga [5, 8, 31]. Infectivity competence, which requires a sufficient parasitaemia, varies spatially and temporally, and in light of previous findings our results, which come from samples collected over a long period of time, must be viewed as results at a point in time and space [5]. Various factors impact upon the infectivity of a host mammal including health, associated parasitic infections, behaviour and contact with vector species, many of which are subject to change as ecosystems suffer anthropogenic damage. More extensive studies of these primates, although challenging for such sylvatic animals that must be protected, would greatly enhance our understanding of transmission cycle dynamics.

Conversely only one Amazonian primate, a member of the *S. libidinosus* species from Estreito municipality in Maranhão State, produced peptide reactions. This was to the TSSApep-III and TSSApep-IV. As cross-reaction between these peptides, which vary by only two amino-acids, has been reported previously it is not possible to say definitively which lineage the animal is infected with, TcIII, TcIV or both [11, 13]. Previous findings strongly suggest TcIV infection in *S. libidinosus*; as far as we are aware it has not previously been isolated from this primate species [3]. This finding of a TcIII/TcIV reacting primate may be supportive of an emerging human disease threat in the Amazon, which could increase with migration into the Amazon, deforestation, land use change and encroachment on primate habitats by human settlement. The lack of further peptide results in the Amazonian primates tested, all of which were either *S. libidinosus* or *A. belzebul*, may be related to lower titre samples, but may also be related to the high level of TcI infection amongst primates in this region. More attempts should be made to design an efficacious TcI specific peptide, for example by comparative genomics, epitope prediction and expression library screening, to seek alternative antigens to TSSA that may be lineage-specific. If necessary, this should be done in conjunction with structural analysis and design of linear peptides that represent conformational epitopes. Peptides should be applicable without avidin-binding to plates to enable inclusion of the few animals that have avidin-binding antibodies. The international surveillance and control programme AMCHA was set up by PAHO in 2004 to tackle the rise in Chagas disease in the Amazon region through surveillance and prevention [9, 20]. It is imperative that the animal reservoir is included in this surveillance. The Amazon region, which was said to be free of TcII, has recently had this lineage isolated from both triatomines and mammals (*Canis familiaris*) in two Amazonian localities, highlighting the need for monitoring of this continually evolving epidemiology [8].

*Leontopithecus rosalia* and *L. chrysomelas* have been shown to be able to maintain long-lasting parasitaemias with TcII [5, 35, 36]. The findings of our study, indicate that both *L. rosalia* and *L. chrysomelas* are viable reservoirs of TcII and TcV/TcVI. Previous studies have shown a high *T. cruzi* seroprevalence in both Amazonian primates (45.5%) and those of the Atlantic Forest (46%) and this implies strong potential for these primates to maintain a variety of lineages within their ecosystems, potentially propagating the parasite to human populations who live increasingly side by side with these primates, which are kept as pets in some regions [34, 35].

These results demonstrate the diversity of *T. cruzi* lineages circulating amongst primates in Brazil in different ecotopes. *Trypanosoma cruzi* has a long evolutionary history with the mammalian wildlife of Latin America leading to the current distribution [39]. However, human actions impact upon the biodiversity of these reservoirs and may have implications for the distribution of the parasite, whether by diluting parasite burden as a result of maintaining biodiversity and thus decreasing disease prevalence, or by maintaining the reservoir and perpetuating the ongoing transmission cycles [40–43].

Pathological changes caused by *T. cruzi* may affect primates, including endangered species such as *L. rosalia, L. chrysomelas* and *L. chrysopygus* [44, 45]. ECG abnormalities as well as elevated cardiac lesion marker serum levels have been seen in free-ranging naturally-infected tamarins [45]. The lack of more severe changes may be due to a lesser pathology produced by the parasite as a result of the longer evolutionary relationship between parasite and primate host, but may also be related to a fitness cost for the animals, causing them to die before these changes are detected, either by predation or by an inability to cope in nature [45]. In the course of conservation programmes wild species are frequently translocated and, in light of the findings here indicative of both TcII and TcV/VI infection in lion tamarins, it is important to do this in a responsible manner to avoid introducing new parasite populations to non-endemic regions [34, 46]. Infections have also been recorded in captive populations, with an active transmission cycle involving *Panstrongylus megistus* being identified in a Brazilian Zoo, which is probably an extension of that of a neighbouring intact forest fragment [46]. This not only highlights the potential for primates to act as sources of infection for humans, but also the potential for sylvatic triatomines to invade other settings where they are in close contact, such as human settlements at forest edges.

The finding of positive reaction with TSSApep-V/VI in both *L. rosalia* and *L. chrysomelas* strongly suggests that the tamarins are a reservoir of hybrid lineages in the Atlantic Forest. We have observed with human samples that only a minor proportion of TSSApep-II/V/VI reactors also react with TSSApep-V/VI, indicating that it is unlikely to be due to a general artefactual cross-reaction between these peptides; we believe this to be in contrast to the situation between TSSApep-IV/TSSApep-III reactivity described herein and elsewhere [11].

Early South American mammals included the Didelphimorphia (opossums) Cingulata (armadillos) and Pilosa (anteaters, sloths); the primates and caviomorph rodents arrived later from Africa between 35 and 55 million years ago [5, 47–49]. A reliance on vicariance biogeography has estimated the time of divergence between New World and Old World trypanosomes to agree with that of the divergence of Gondwanan landmasses [50]. However new theories have arisen proposing that trypanosomes in the New World may have reached South America much later, based on the diversity of the *T. cruzi* clade, and surmise that a viable route of dispersion may have been via bats which are known to be hosts of a wide range of trypanosomes and are able to cross large bodies of water [50]. Hamilton et al. [50] suggest that the ancestor of *T. cruzi* and *T. rangeli* originated in bats and was then seeded to South America via bat dispersion. However, with growing evidence of dispersal of other species across dispersion barriers such as the Atlantic Ocean there is potential for other mammals, such as primates and caviomorph rodents to have aided in this seeding. Primates are thought to have made their long-distance journey approximately 40 million years ago making this a viable option [47].

## Conclusions

We have shown this ELISA to successfully identify lineage-specific reaction even when haemoculture has been negative, and even in one instance when IFAT serology was also negative. The assay can be applied to sylvatic primates and is a useful tool for understanding the eco-epidemiology of the various lineages of *T. cruzi* where isolation and genotyping are severely limited. In particular, it indicates that the test may identify as yet undetected reservoirs of TcV and TcVI and further our understanding of the sylvatic cycles of these lineages. As all mammal species are considered to be susceptible to *T. cruzi* infection there would be great value in expanding the test to a wide range of species in order to elucidate novel hosts. Future research is required in order to refine this test in other species, as well as identifying a TcI specific epitope that could be applied as a lineage-specific peptide. Commercially available secondary antibodies allow the ELISA to be applied to a greater variety of species, however often the phylogenetic relationship of species available is not as close as is ideal. It may be desirable to use a pan-generic conjugate such as Protein G, and to adapt the assay to a rapid diagnostic test for point-of-capture application to animals in the field (work in progress). With greater knowledge of sylvatic transmission cycles there may be increased potential for predicting outbreaks, particularly at the interface between human activity and sylvatic mammalian habitats where new cases are frequently seen, and producing sustainable targeted control measures. Collaboration by all those involved in studying aspects of the parasite, its ecology and the dynamic social context within which it sits will enhance this work.

## Abbreviations

DALY: Disability-adjusted life year
GPI: Glucose-6-phosphate isomerase
HSP60: Heat shock protein 60
IFAT: Indirect fluorescent antibody test
PCR: RFLP: Polymerase chain reaction: restriction fragment length polymorphism
SNP: Single nucleotide polymorphism
Tc: *Trypanosoma cruzi*
TSSA: Trypomastigote small surface antigen
TSSApep: Lineage-specific TSSA peptide
